# Effectiveness of chronic care models: opportunities for improving healthcare practice and health outcomes: a systematic review

**DOI:** 10.1186/s12913-015-0854-8

**Published:** 2015-05-10

**Authors:** Carol Davy, Jonathan Bleasel, Hueiming Liu, Maria Tchan, Sharon Ponniah, Alex Brown

**Affiliations:** 1 grid.430453.5South Australian Health & Medical Research Institute, Adelaide, South Australia Australia; 2 grid.415508.d0000000119646010The George Institute for Global Health, Camperdown, New South Wales Australia

**Keywords:** Chronic care model, Integrated care, Chronic disease, Primary healthcare

## Abstract

**Background:**

The increasing prevalence of chronic disease and even multiple chronic diseases faced by both developed and developing countries is of considerable concern. Many of the interventions to address this within primary healthcare settings are based on a chronic care model first developed by MacColl Institute for Healthcare Innovation at Group Health Cooperative.

**Methods:**

This systematic literature review aimed to identify and synthesise international evidence on the effectiveness of elements that have been included in a chronic care model for improving healthcare practices and health outcomes within primary healthcare settings. The review broadens the work of other similar reviews by focusing on effectiveness of healthcare practice as well as health outcomes associated with implementing a chronic care model. In addition, relevant case series and case studies were also included.

**Results:**

Of the 77 papers which met the inclusion criteria, all but two reported improvements to healthcare practice or health outcomes for people living with chronic disease. While the most commonly used elements of a chronic care model were self-management support and delivery system design, there were considerable variations between studies regarding what combination of elements were included as well as the way in which chronic care model elements were implemented. This meant that it was impossible to clearly identify any optimal combination of chronic care model elements that led to the reported improvements.

**Conclusions:**

While the main argument for excluding papers reporting case studies and case series in systematic literature reviews is that they are not of sufficient quality or generalizability, we found that they provided a more detailed account of how various chronic care models were developed and implemented. In particular, these papers suggested that several factors including supporting reflective healthcare practice, sending clear messages about the importance of chronic disease care and ensuring that leaders support the implementation and sustainability of interventions may have been just as important as a chronic care model’s elements in contributing to the improvements in healthcare practice or health outcomes for people living with chronic disease.

**Electronic supplementary material:**

The online version of this article (doi:10.1186/s12913-015-0854-8) contains supplementary material, which is available to authorized users.

## Background

Chronic diseases have a substantial impact on the lives of people living in both developed and developing countries. Of the 57 million deaths in 2008, 36 million (63%) were a direct result of chronic diseases, principally cardiovascular disease, diabetes, cancer and chronic respiratory diseases. Nine million of these deaths occurred in people under 60 years of age and ninety per cent of these premature deaths occurred in low- and middle-income countries [1]. It is also the case that disadvantaged and marginalised communities in developed countries suffer an increasing burden of chronic disease [2].

As a way of combating this growing health crisis, researchers have attempted to develop comprehensive strategies to manage chronic disease and to deliver improved chronic disease care. The primary aim of many integrated care or chronic disease management programs is to reduce fragmentation while at the same time improving health outcomes at an acceptable cost to the healthcare system [3,4]. Many of the current chronic disease management strategies were first identified by MacColl Institute for Healthcare Innovation at Group Health Cooperative, commonly referred to as the Wagner chronic care model (Wagner CCM), which was based on six key elements [5-7]. These elements focus on mobilising community resources, promoting high quality care, enabling patient self-management, implementing care consistent with evidence and patient preferences, effectively using patient/population data, cultural competence, care coordination, and health promotion [8]. Yet while the broad elements may be similar to the Wagner CCM developed by the MacColl Institute for Healthcare Innovation, what constitutes a CCM and how it is implemented and delivered within healthcare services, has continued to evolve [9,10].

A number of systematic literature reviews have already focused on which of the elements or combination of elements included within a CCM were effective in improving healthcare practice and health outcomes. One of the first systematic literature reviews to include all six elements of the Wagner CCM focused on the provision of care to chronic obstructive pulmonary disease (COPD) [11]. While the review found that the implementation of two or more elements was likely to reduce healthcare usage by COPD patients, the authors also identified significant heterogeneity between the ways in which each of the elements were implemented. Another systematic literature review [12] looked at the association between improved performance and the implementation of integrated quality management models which included a CCM. Again, there was some evidence that implementing interventions based on a CCM improved performance and health outcomes. Other systematic reviews have identified small to moderate improvements in health outcomes associated with diabetes [13], improved adherence to inhaled corticosteroids among asthmatics [14], and improvements to mental and physical health outcomes for patients with mental disorders such as depression [15]. Pasricha et al [16] also conducted a systematic literature review focusing on effectiveness of two of the elements included within the Wagner CCM - decision support and clinical information systems. These authors found that the implementation of either or both elements resulted in modest improvements to care provided for people living with HIV.

These previous systematic reviews have tended to focus on effectiveness for improving health outcomes. They have also limited their inclusion criteria to evidence from randomised [11,14,15] and/or non-randomised trials, cross sectional studies and cohort studies [13,16]. This systematic literature review broadens the work of other reviewers in two ways. First, it focuses on healthcare practice as well as the health outcomes associated with implementing a CCM. This is particularly important as the quality of healthcare practice is a key determinant of health outcomes for patients [17]. Improvements to healthcare practice not only benefit the patients in terms of improved health outcomes but also ensure considerable savings to the healthcare system [18].

The second feature of this systematic literature review is that case series and case studies have also been included. To our knowledge only one other systematic literature review has included case studies [12]. While the main argument for excluding this type of literature is that they are not of sufficient quality or generalizability, case studies and case series have been included on the basis of completeness. Rather than dismissing any study based on methodology alone, we have instead focused on presenting information about the quality of these and other featured studies.

## Method

### Review objective and questions

The objective of this systematic literature review was to identify and synthesise relevant international evidence on the effectiveness of CCMs elements for improving healthcare practices and health outcomes. The questions asked by this review were:

1. What elements of a CCM have been implemented into a PHC setting?
2. Do the identified elements improve healthcare practices delivered to patients living with chronic disease?
3. Do the identified elements improve the health outcomes of patients living with chronic disease?

### Inclusion criteria

#### Types of participants

This review considered studies that either focused on people with or healthcare providers that cared for people with a non specific chronic disease or alternatively with at least one of the following specific chronic diseases - cardiovascular disease, chronic kidney disease, chronic respiratory disease, type 2 diabetes mellitus, depression and HIV/AID -) in a primary healthcare setting.

Primary healthcare is generally defined as first-contact, accessible, continued, comprehensive and coordinated healthcare provided by a single practitioner (e.g. GP, nurse practitioner) or a multidisciplinary team of professionals in a community practice. For the purposes of this review however, primary healthcare is first-contact, accessible, continued, comprehensive and coordinated care. First-contact care is accessible at the time of need; on-going care focuses on the long-term health of a person rather than the short duration of the disease; comprehensive care is a range of services appropriate to the common problems in the respective population and coordination is the role by which primary care acts to coordinate other specialists that the patient may need [19]. Primary healthcare also includes primary care settings that have only one health professional, i.e. a general practitioner.

#### Elements of a chronic care model

In order to identify elements that should be included as part of this review, a scoping exercise of published chronic care models was undertaken. This scoping exercise identified two additional key elements - case management [20] and family support [21] which had previously been included as part of a chronic care model, bringing the total number of elements included within this review to eight. Studies which had implemented at least two of the these eight elements were included in this review:

1. Facilitated community support (CS) to meet the needs of patients
2. Facilitated unpaid/informal family support (FS) to meet the needs of patients
3. Self-management support (SMS) to meet the needs of patients
4. Health system (HS) improvement to meet the needs of health-care providers
5. Delivery system design (DSD) to meet the needs of health-care providers
6. Enhanced health care professional case management (CM) support to meet the needs of patients
7. Decision support (DS) to meet the needs of health-care providers
8. Clinical information systems (CIS) to meet the needs of health-care providers

#### Types of outcome measures

In addition to describing the elements included within a CCM, outcome measures for effectiveness included any reported changes (improvements or declines) to healthcare practice, or the health outcomes of patients as a result of the implementation of a CCM.

#### Types of studies

This review focused on quantitative (e.g. randomised and non-randomised control trials, cross-sectional and cohort studies, case studies and case series) and qualitative studies.

### Search strategy

Seven electronic databases (MEDLINE, Cinahl, Embase, Informit Online, PsycINFO, Scopus, and Web of Science) were searched for articles published in English language between January 1998 to April 2013 and met the above inclusion and exclusion criteria. The Medline search strategy is provided in [see Additional file 1] was originally set up in MEDLINE and then modified for the other databases.

### Study selection

Four authors (CD, HL, MT, SP) were involved in study selection. For each paper, two of these authors independently scanned the identified studies and excluded studies according to the criteria above, on the basis of titles and abstracts. Full text copies of the papers deemed to meet the inclusion and exclusion criteria were these retrieved and two of the review authors reviewed these publications. Authors of relevant papers were contacted if the full text article were not available. If there was uncertainty or disagreement, consensus was reached by discussion and consultation with the review authors.

### Bias appraisal

Four authors (CD, HL, MT, SP) were also involved in the Bias Appraisal. Two of these authors independently assessed the risk of bias onall of the papers included in this review. The Cochrane Handbook for Systematic Reviews of Interventions was used to assess bias for randomised and non-randomised control trials, cross-sectional and cohort studies [22]. The Joanna Briggs critical appraisal tool was used to measure the bias of case studies and case series [23]. As the first objective of the review was to identify elements of a CCM which have been included in studies, and then identify the effectiveness of these elements for improving health outcomes and the provision of healthcare, studies were not excluded based on this appraisal [see Additional file 1: Table S1–S5].

### Data extraction

Data was extracted from primary studies and included in pre-defined data extraction tables by the four review authors (CD, JB, HL, MT). The extracted data included specific details about the geographical context, study methods and disease focus [see Additional file 1: Table S6], elements included in the intervention, study participants, and outcomes of significance to the review questions [see Additional files 1: Table S7–Table S11]. Data has been presented in narrative form including tables and figures to aid in data presentation where appropriate.

## Results

### Literature search

**Figure 1:**
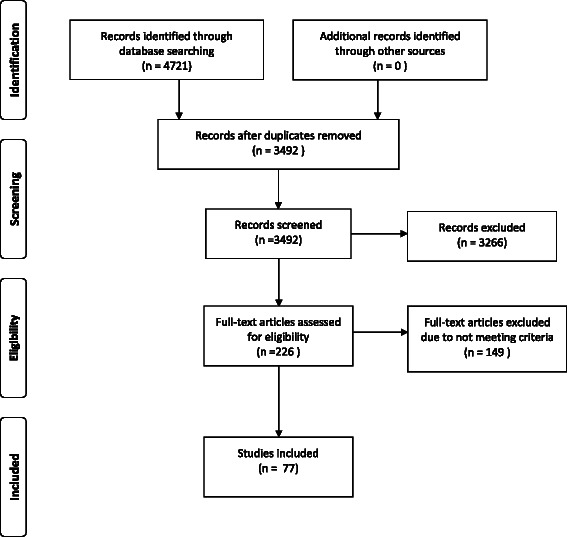
PRISMA 2009 Flow Diagram.

The search of information sources returned 3492 articles from the initial searches of electronic databases. The majority of these studies were subsequently excluded based on their title or abstract because they clearly did not meet the inclusion criteria for this review. A total of 226 full text articles were obtained and a further 149 were excluded as they also did not meet the inclusion criteria. This resulted in the inclusion of 77 published peer-reviewed papers which were ultimately included in this review (Figure 1).

### Description of chronic care models

The majority of studies were conducted in the Americas, including United States of America, Canada and Mexico [24-76]. A number of studies were also conducted in Europe including United Kingdom, Spain, Belgium, Italy, Denmark, Netherlands and Germany [20,77-88]. A further six studies were conducted in Australia and New Zealand [21,89-93], one study was conducted in Taiwan [94], one in the United Arab Emirates [95] and one in South Africa [96] [see Additional file 1: Table S6].

The majority of studies focused on the provision of care for diabetes [21,24,26,27,29,31,33-38,40-42,44-53,55-60,62,65-70,72,75,76,79,80,83-87,90,91,93,94,96]. Included studies also focused on cardiovascular disease [20,25,28,30,32,39,43,54,61,63,64,71,73,88,89,91,95], depression [34,51,53,74,76,90], respiratory disease [90], including chronic obstructive pulmonary disease [77,81,82,92,93], and renal disease [21,90]. Other studies [20,25,28,30,32,39,43,54,61,63,64,71,73,88,89,91,95] focused on the provision of care to patients with chronic diseases more generally [see Additional file 1: Table S6].

While a range of CCM elements were used across the papers reviewed, the most commonly used element was SMS, while only two papers included FS (Table 1). However, there was substantive variation between studies in both the combination of included elements and also in how these elements were implemented. For example, descriptions of SMS implemented in primary care settings included development of care guides and individualised patient action plans [25,28,92], individual counselling or coaching, [25,42,74,97], education programs on disease management [28,31,33,51,58,62,74,77,86,89,98,99], programs on empowerment, goal-setting and motivation [26,42,51,58,79,92], and use of support groups [62,98,99]. Descriptions of how other CCM elements were implemented also differed substantially between studies, meaning that between study changes to healthcare practice and health outcomes as a result of implementing CCM elements were not easily comparable.

**Table 1:** **Overview of CCM Elements Reviewed**

**Element**	**Number of Papers**
Self-Management Support	50
Delivery System Design	39
Clinical Information Systems	37
Decision Support	36
Case Management	19
Health System	13
Community Support	13
Family Support	2
CCM - Elements Not Specified	4

### Effectiveness of chronic care models

To explore the effectiveness of CCM elements, the review focused on the analyses of randomised controlled trials (RCTs), non-randomised controlled trials (non-RCTs), retrospective cohort studies, as well as case studies and case series. Measures of effectiveness relating to health outcomes relevant to specific chronic diseases (e.g. improvements to HBA1c for diabetes) as well as healthcare practice appropriate to the management of chronic disease (e.g. concordance with clinical guidelines), were reported by 63 of the 77 studies included in this review.

In a small number of studies [35,70,75] the Assessment of Chronic Illness Care (ACIC) was used to assess level of implementation of CCM elements in primary healthcare settings. The ACIC contains 28 items across the six elements within the Wagner CCM: CS, HS, SMS, DS, DSD and CIS, with each assigned a numeric score from 0 to 11. Individual providers or healthcare teams were asked to rate level of implementation through self-report. The equivalent patient self-reporting tool (Patient Assessment of Care for Chronic Conditions) was also used in three studies [47,66,87] to measure quality of healthcare based on five sub-scales: patient activation, DSD, DS, goal setting, problem solving/contextual counselling and follow up/coordination.

Findings pertaining to the quality of the included papers and the reported effectiveness associated with specific CCM elements for improving health outcomes and healthcare practices are presented below by study type.

#### Randomised controlled trials

Of the 13 RCTs that measured the efficacy of a CCM as defined by our search criteria, the majority (n = 8) were conducted in the USA [25,26,28,42,51,62,69,74]. Control groups generally either received usual care or received less intensive intervention. Six studies focused on diabetes [24,26,42,62,69,79], two studies on COPD [77,92], one study on depression [51], and four studies on non-specific chronic disease or multi-morbidity [20,25,28,74].

A significant potential risk of bias was identified in many of the included RCT papers [see Additional file 1: Table S1]. Of particular concern was the risk of detection bias which was assessed as either high or unclear for all but two of the papers [69,74].

Findings of significant healthcare practice or health outcome improvements associated with CCM interventions were inconsistent [Additional file 1: Table S7]. While many studies reported significant changes in health outcomes from baseline in the intervention group, significant between-group differences were often lacking [42,92], and a number of studies reported no intervention effect for any health outcome [26,28,51,69,74]. Randomised control trials that reported significant changes in health outcomes from baseline for the intervention groups had implemented the following elements:

- SMS [62,77]
- DSD [62]
- CIS [77]
- DS [62]
- CM [24,77]
- HS [24]

Two RCTs reported on healthcare practice change. One reported a significant improvement in monitoring of symptoms and risk factors was associated with CM and HS [24], while the second study identified a deterioration in patient education [77].

#### Non-randomised control trials

Two non-RCT papers were also reviewed, one conducted in the USA focusing on COPD [32] and the other in Europe which focused on chronic disease more generally [97]. Only one of these studies looked at effectiveness [see Additional file 1: Table S7], demonstrating significant reductions in mortality in an intervention group referred to a nurse care manager equipped with specialised information and IT tools, however findings were not significant after two year follow up [32]. The second study evaluated implementation of CIS, SMS and DSD elements into primary healthcare practices and reported on the proportion of elements that had been implemented at two year follow up [97]. While reporting bias was low, the risk of selection, sampling, detection and attrition bias for non-RCT papers was considered to be, at best unclear, if not high [see Additional file 1: Table S2].

#### Retrospective cohort studies

All six observational cohort studies were conducted retrospectively using chart reviews of electronic patient health records, registries and patient databases to evaluate CCM elements implemented at a practice or practice group level. Four of the studies were conducted in the USA [31,33,49,61] and two were conducted in Europe [83,85]. Five studies focused on diabetes [31,33,49,83,85] and one focused on non-specific chronic disease risk factors [61].

The risk of selection and sampling bias was assessed as high or unclear for all but one study [31]. Likewise, the risk of detection bias was also considered to be high or unclear for all but one other study [61] [see Additional file 1: Table S3].

Three of the studies reported improvements to healthcare practice as well as health outcomes for diabetic patients [31,33,85] while one study [61] only reported on improvements to health outcomes for diabetic patients [see Additional file 1: Table S9]. Improvements were found to be associated with the following CCM elements.

- SMS [31,33,85]
- DSD [31,33,83,85]
- CIS [31,33,83]
- DS [31,33,83,85]
- CM [31,33]

#### Cross-sectional studies

Of the 11 cross-sectional studies identified in this review, all but one study [91] included elements implemented to support diabetic care. In addition, only two studies [87,91] were conducted outside of the USA.

Eight of the 11 cross-sectional studies [13,35,40,45,46,66,70,87] either did not have sufficient information to make an assessment, or were considered to be at high risk of selection bias. Only four of the cross-sectional studies met the criteria for being at low risk of detection [45-47,70] or attrition biases [35,46,60,70], while seven were assessed as low risk for reporting bias [35,45-47,60,75,87] [see Additional file 1: Table S4].

Three of the cross-sectional papers reported associations between implementation of CCM elements and improvements to clinical outcomes, [35,66,91] with one study reporting improvement in clinical outcomes and healthcare practice [60] [see Additional file 1: Table S10]. Improvements were found to be associated with the following CCM elements.

- SMS [35,60,66,91]
- DSD [35,60,66,91]
- CIS [35,60,66,91]
- DS [35,60]
- CM [66]
- HS [35,91]
- CS [35]

#### Case studies and case series

Similar to papers presented above, the vast majority of case studies and case series (25 of 31 papers) included diabetic patients when assessing the effectiveness of CCMs for improving health outcomes of, or health care practice [21,27,29,36-38,44,48,50,53,55-57,65,67,72,76,84,90,91,93,94,96]. The majority of these case studies and case series (20 of 31 papers) were conducted in USA [27,29,34,36-38,44,48,50,53-57,63,65,67,72,76].

None of the case studies and case series papers included in this review met all of the nine critical appraisal criteria defined by the Joanna Briggs Institute [23]. Of particular concern was that nine of these studies did not sufficiently define the inclusion criteria, and only two of the 31 papers identified confounding factors [Additional file 1: Table S5].

Twenty two of the case studies or case series papers reported associations between improved health outcomes [21,29,34,36-38,44,48,54,63,65,67,72,78,84,90,91,93-95] and the implementation of SMS [see Additional file 1: Table S11]. In addition, associations were also found for improved health outcomes and the implementation of the following elements.

In addition, associations were also found for improved health outcomes and the implementation of the following elements.

- DSD [21,29,36,37,54,65,67,72,84,95]
- CIS [21,29,37,54,65,72,76,95]
- DS [29,36,37,44,65,67,72,76,93,95]
- CM [29,34,36,44,78,90]
- HS [38]
- CS [21,29,48]
- FS [21]

Two case studies [67,95] found an association between implementing CCM elements and a decline in a health outcome (decreased high-density lipoprotein and increased low-density lipoprotein respectively). However, out of 77 papers included within this review, these were the only studies to report a negative health outcome associated with the implementation of CCM elements.

Twenty five of the case studies or case series [21,27,29,30,36-38,44,48,50,53-57,67,73,78,81,84,93-96] reported an association between improved healthcare practices and the implementation of the following elements.

- SMS [21,27,29,30,36-38,44,48,50,54-57,67,78,81,84,93,95]
- DSD [21,27,29,36,37,50,54,56,67,81,84,94-96]
- CIS [21,27,29,30,37,50,54-57,81,95]
- DS [27,29,93,95]
- CM [29,36,44,78]
- HS [27,38,96]
- CS [21,27,29,48,50,94]
- FS [21]

Only one case study [57] out of the 77 papers included within this review suggested an association between implementing elements of a CCM and a decline in healthcare practices (documentation).

## Discussion

Of the papers which did include measures of effectiveness, the majority found an association between the implementation of CCM elements and improvements with healthcare practice or health outcomes for people living with chronic disease. Only two papers [67,95] reported association between implementing CCM elements and a decline in any of the health outcomes measured (decreased high-density and increased low-density lipoproteins respectively), while one paper [57] suggested an association between the implementation of CCM elements and a decline in healthcare practices (documentation).

One of the primary findings of this systematic literature review was considerable study variability, both in the combination of and ways in which CCM elements were implemented. For this reason it was impossible to clearly identify any optimal combination of the eight CCM elements that could lead to improvements in either healthcare practice or health outcomes. A direct relationship between any combination of CCM elements and improvements to either healthcare practice or health outcomes was further placed into doubt by the RCT studies that compared outcomes from the implementation of two different combinations of CCM elements [38,44]. Despite differences in the combination of elements included, researchers were unable to find any significant variation in outcomes. Similarly, studies that focused on the implementation of self-selected elements across multiple sites found very little between site differences in either the type or strength of healthcare practice or health outcome improvements [50,57,78,95]. This suggests that factors other than or in addition to the implementation of CCM elements may play a role in improving healthcare practices and health outcomes for people living with chronic disease [100].

One of the benefits of including case studies in this systematic review was that they tended to provide a more detailed account of how CCM elements were implemented. Of the 19 case studies that described these processes in more detail, eight specifically utilised the Plan-Do-Study-Act cycle [27,37,54,65,72,93,95,101], while a further five developed various learning collaboratives [29,50,53,57,76] as part of the development and implementation process. One of the key findings of these studies was that Plan-Do-Study-Act cycles and learning collaboratives appeared to be associated with the development of contextually relevant interventions. In addition, these methods often meant that the healthcare providers involved in the implementation process were engaged with development, encouraging a sense of ownership and consequently responsibility for the success of the intervention. The authors of these papers also described how healthcare providers who were involved in the development process had an opportunity to reflect on, gaining for example, a more nuanced understanding of how the care they provided could address the needs and priorities of the communities they served.

Reflective practice is a key component for developing clinical knowledge and skills [102] and can, in and of itself, lead to significant improvements in healthcare by assisting to bridge the gap between theory and practice [103,104]. Importantly for the implementation of interventions including CCM elements, reflective practice also encourages healthcare providers to identify anomalies between the ways in which they currently practice and organisational priorities for the future [105]. Within a healthcare setting, this involves analysing one’s own experiences and modifying behaviour based on these reflections in order to improve the way in which healthcare is provided. While not without some challenges, an individual’s reflective practice is enhanced when there is an opportunity to work with others in a group setting [106]. The methods, including the Plan-Do-Study-Act cycles and learning collaboratives described in this systematic review, can assist this process by developing collegial environments within which this reflective group practice can occur.

Although not specifically addressed by papers in this review, spending the time and resources to develop and implement a CCM may have also underpinned both healthcare practice and health outcome improvements by signalling to staff that improving chronic disease care was a priority for their healthcare service. Yet simply communicating these messages may not be sufficient to ensure improvement. What was evident in a number of papers, was the key role that leaders played in guiding the development and implementation process. Once started, leaders within these organisations needed to be committed to the implementation and sustainability of a new CCM [27,31,43,52,54,71,72,93]. As was highlighted in the Wagner CCM under HS [107], without this commitment, any improvements to either health outcomes or healthcare practices were likely to have been lost [43,52].

Providing a collegial environment which supports reflective practice, sending clear messages about the importance of chronic disease care and ensuring that leaders support the implementation and sustainability of interventions appear to contribute to the health outcomes and healthcare practices identified in papers included in this review. However, this list is by no means complete and further work is required to identify other facilitators and barriers which could influence the implementation of similar interventions. However, the findings in this systematic literature review do suggest that other models of care, including alternatives to CCM elements included in this review could be equally successful in improving the health outcomes and healthcare practices within primary healthcare services, particularly when they address the particular needs of patients within each context [95].

Contextual relevance is especially important given that although the burden of chronic disease is highest within disadvantaged populations, the majority of studies which have implemented the eight CCM elements included in this review have focused on interventions within advantaged populations living in developed countries [see Additional file 1: Table S6]. In particular, FS which was the least utilised CCM element (Table 1) may be particularly useful within, for example, Aboriginal peoples living with chronic disease [21]. Whether this or any other CCM elements can help to improve healthcare practices and health outcomes for disadvantaged populations more generally is not as clear. Outcomes from this review suggest that targeted approaches whereby leaders provide clear direction and support [108] and also encourage healthcare practitioners to reflect on how their own practices may need to change to meet the needs of particular populations are more likely to stimulate improvements to health outcomes and healthcare practice.

### Limitations

There are a number of limitations to this review. Of particular concern was the high risk of bias in the RCT, non-RCT, retrospective cohort and cross sectional studies. In addition, the quality of the case studies included in this review was considered to be poor. In addition, as previously noted the interventions differ from one study to another, meaning that generalizations were impossible to make and which suggestions based on existing evidence have been made for why a CCM might lead to improved healthcare process and health outcomes these are yet to be tested.

## Conclusions

The key finding from this systematic literature review was the wide variability between the elements included within CCMs and the way in which these elements were implemented. While the majority of papers reported improvements to either healthcare practice or health outcomes as a result of implementing a CCM, it was not possible to identify which elements or combination of elements led to these improvements. Rather these results suggested that factors other than or in addition to the implementation of CCM elements may play a role. While not exclusive, these may include collegial environments which support reflective practice, sending clear messages about the importance of chronic disease care and ensuring that leaders support the implementation and sustainability of interventions. Given the high prevalence of chronic disease in disadvantaged populations including Indigenous communities, elements including FS could play a greater role in improving the management of and outcomes from chronic disease for these peoples.

## Additional file

Additional file 1:
**Search strategy. Table S1.** RCT risk of bias. **Table S2.** Non-RCT risk of bias. **Table S3.** Retrospective cohort study risk of bias. **Table S4.** Cross sectional study risk of bias. **Table S5.** Case study and case series quality appraisal. **Table S6.** Description of chronic care models. **Table S7.** RCT study outcomes. **Table S8.** Non-RCT study outcomes. **Table S9.** Retrospective study outcomes. **Table S10.** Cross sectional study outcomes. **Table S11**. Case study and case series outcomes.

## Abbreviations

ACIC: Assessment of chronic illness care
CM: Case management
CCM: Chronic care model
COPD: Chronic obstructive pulmonary disease
CIS: Clinic information system
CS: Community support
DS: Decision support
DSD: Delivery system design
S: Family support
HS: Health system
RCTs: Randomised control trials
SMS: Self-management support
