# Supplementary material for: Effectiveness of chronic care models: opportunities for improving healthcare practice and health outcomes: a systematic review
Source: BMC Health Serv Res. 2015 May 10;15:194. doi: 10.1186/s12913-015-0854-8 (PMC4448852; doi:10.1186/s12913-015-0854-8)
Supplement: Additional file 1: — Search strategy. Table S1. RCT risk of bias. Table S2. Non-RCT risk of bias. Table S3. Retrospective cohort study risk of bias. Table S4. Cross sectional study risk of bias. Table S5. Case study and case series quality appraisal. Table S6. Description of chronic care models. Table S7. RCT study outcomes. Table S8. Non-RCT study outcomes. Table S9. Retrospective study outcomes. Table S10. Cross sectional study outcomes. Table S11. Case study and case series outcomes. [file 12913_2015_854_MOESM1_ESM.docx]

# Additional File 1 – Search Strategy

1. "Chronic care model*".tw.

2. (Chronic care adj5 model*).tw.

3. (Model* adj5 (collaborative adj5 care)).tw.

4. (Chronic care adj5 framework*).tw.

5. (Chronic disease adj5 care).tw.

6. (Chronic illness adj5 care).tw.

7. (Model* adj2 care).tw.

8. (Wagner* adj5 model*).tw.

9. (Wagner* adj5 chronic care model*).tw.

10. (Flinder* adj5 model).tw.

11. (Stanford* adj5 model).tw.

12. "delivery of health care"/ or "delivery of health care, integrated"/

13. models, organizational/

14. patient care management/

15. (Model* adj3 care).tw.

16. 1 or 2 or 3 or 4 or 5 or 6 or 7 or 8 or 9 or 10 or 11 or 12 or 13 or 14 or 15

17. "Primary health care".mp.

18. "Primary care".tw.

19. (Primary health adj5 service).tw.

20. exp General Practice/

21. comprehensive health care/ or exp primary health care/

22. community health services/ or community health nursing/ or community mental health services/

23. preventive health services/ or exp health education/

24. 17 or 18 or 19 or 20 or 21 or 22 or 23

25. Chronic Disease/

26. cardiovascular diseases/ or cardiovascular abnormalities/ or cardiovascular infections/ or heart diseases/ or vascular diseases/

27. renal insufficiency, chronic/ or exp kidney failure, chronic/

28. chronic kidney disease.tw.

29. heart disease*.tw.

30. cardiovascular disease*.tw.

31. exp Pulmonary Disease, Chronic Obstructive/

32. chronic respiratory disease*.tw.

33. exp Diabetes Mellitus, Type 2/

34. (diabetes adj5 care).tw.

35. Depression/

36. depression.tw.

37. (depressive or dysthym*).mp.

38. depressive disorder/ or depressive disorder, major/ or dysthymic disorder/

39. exp HIV/

40. 25 or 26 or 27 or 28 or 29 or 30 or 31 or 32 or 33 or 34 or 35 or 36 or 37 or 38 or 39

41. 16 and 24 and 40

42. limit 41 to (humans and yr="1998 -Current")

# Additional File 2 – RCT Risk of Bias

| **First AUTHOR (Year)** | **SELECTION BIAS** | **PERFORMANCE BIAS** | **DETECTION BIAS** | **ATTRITION BIAS** | **REPORTING BIAS** | **OTHER BIAS** |
| --- | --- | --- | --- | --- | --- | --- |
| Barcelo (2010) | High | High | Unclear | N/A | High | No |
| Boyd (2010) | Unclear | Unclear | High | Low | Unclear | No |
| Casas (2006) | Low | High | Unclear | Low | Low | Yes |
| Chin (2007) | High | Unclear | Unclear | Low | Low | Yes |
| Coleman (1999) | Low | High | High | Low | Low | No |
| Olivaius (2001) | Low | Low | Unclear | Low | Low | No |
| Hiss (2007) | Unclear | Low | Unclear | Unclear | Low | No |
| Landis (2007) | Low | High | High | Unclear | Low | No |
| Martin (2012) | Low | Low | High | Low | Low | Yes |
| Piatt (2006) | Low | Unclear | Unclear | Low | Low | No |
| Rea (2004) | Low | High | High | Low | Low | No |
| Smith (2008) | Low | Low | Low | Low | Low | No |
| Wolff (2009) | Unclear | Unclear | Low | Unclear | Low | No |

# Additiona File 3 – Non-RCT Risk of Bias

| FIRST AUTHOR (YEAR) | SELECTION BIAS | SAMPLING BIAS | DETECTION BIAS | ATTRITION BIAS | REPORTING BIAS | OTHER BIAS |
| --- | --- | --- | --- | --- | --- | --- |
| Dorr (2008) | Unclear | High | High | Unclear | Low. | No |
| Meulepas (2007) | High | Unclear | Unclear | High | Low | No |

# Additional File 4 – Retrospective Cohort Study Risk of Bias

| **FIRST AUTHOR (YEAR)** | **SELECTION BIAS** | **SAMPLING BIAS** | **DETECTION BIAS** | **ATTRITION BIAS** | **REPORTING BIAS** | **OTHER BIAS** |
| --- | --- | --- | --- | --- | --- | --- |
| Dipiero (2008) | Low | Low | High | Low | Low | No |
| Dorr (2005) | Unclear | Unclear | High | Low | Low | No |
| Kimura (2008) | Unclear | Unclear | Unclear | N/A | Unclear. | No |
| Mohiddin (2006) | Unclear | High | High | Low | Low risk | No |
| O’Toole (2010) | High | High | Low | Unclear | Unclear | No |
| Sunaert (2010) | High | High | Unclear | Low | Unclear | No |

# Additional File 5 – Cross-Sectional Studies Risk of Bias

| **FIRST AUTHOR (YEAR)** | **SELECTION BIAS** | **SAMPLING BIAS** | **DETECTION BIAS** | **ATTRITION BIAS** | **REPORTING BIAS** | **OTHER BIAS** |
| --- | --- | --- | --- | --- | --- | --- |
| Feifer (2001) | High | Low | Unclear | Low | Low | No |
| Grossman (2008) | High | Low | High | N/A | Unclear | No |
| Hung (2008) | High | Low | Low | N/A | Low | Yes |
| Jackson (2005) | Unclear | Low | Low | Low | Low | No |
| Jackson (2008) | Low | Unclear | Low | High | Low | Yes |
| Nutting (2007) | Low | Low | High | Low. | Low | No |
| Schmittdiel (2007) | High | High | Unclear | High | Unclear | No |
| Si (2008) | Unclear | Unclear | Unclear | N/A | Unclear | No |
| Strickland (2010) | Unclear | Unclear | Low | Low | Unclear | No |
| Szecsenyi (2008) | Unclear | High | High | High | Low | No |
| Wong (2013) | Low | Unclear | Unclear | Low | Low | No |

# Additional File 6 – Case Study and Case Series Quality

| **FIRST AUTHOR (YEAR)** | Q1* | Q2* | Q3* | Q4* | Q5* | **Q6*** | **Q7*** | **Q8*** | **Q9*** |
| --- | --- | --- | --- | --- | --- | --- | --- | --- | --- |
| Baynouna (2010) | No | Yes | No | Yes | N/A | Yes | N/A | Yes | Yes |
| Chin (2004) | Yes | Yes | No | Yes | N/A | Yes | No | Yes | Yes |
| Chiou (2001) | Unclear | Unclear | No | Yes | N/A | N/A | N/A | Yes | Yes |
| Ciccone (2010) | No | Yes | No | Yes | N/A | Yes | No | Yes | Yes |
| Daniel (2004) | No | No | No | Yes | No | Unclear | N/A | Unclear | Unclear |
| Delon (2009) | No | No | No | Unclear | Unclear | Unclear | N/A | Unclear | Unclear |
| Esperat (2012) | No | Yes | No | Yes | N/A | Yes | Yes | Yes | Yes |
| Friedman (1998) | No | No | No | Yes | N/A | Yes | No | Unclear | Unclear |
| Gabbay (2011) | No | Yes | No | Yes | N/A | Yes | N/A | Yes | Yes |
| Glasgow(2002) | No | Yes | Yes | No | No | Yes | Unclear | Yes | Unclear |
| Harvey (2008) | No | yes | No | No | N/A | Yes | No | No | Yes |
| Huckfeldt (2012) | No | No | No | Yes | No | Unclear | N/A | Unclear | Unclear |
| Jenkins (2011) | No | Yes | No | Yes | N/A | Yes | N/A | Yes | Yes |
| Katz (2009) | N/A | Yes | No | Yes | N/A | Yes | No | Yes | Yes |
| Landis (2006) | No | Yes | No | Yes | No | Yes | N/A | Unclear | Unclear |
| Lemmens (2009) | No | Yes | No | Yes | N/A | Yes | No | Yes | Yes |
| Letourneau (2006) | No | Yes | No | Yes | N/A | Yes | No | Unclear | Unclear |
| Lyon (2011) | No | Unclear | No | Unclear | N/A | Unclear | N/A | Unclear | Unclear |
| McCulloch (1998) | Yes | Yes | No | Yes | Yes | Yes | N/A | Yes | Yes |
| McCulloch (2000) | No | Yes | No | Yes | N/A | Yes | No | Yes | Unclear |
| McRae (2008) | N/A | Yes | No | Yes | N/A | Yes | N/A | Yes | Yes |
| Montori (2002) | No | Yes | No | Yes | No | Yes | N/A | Yes | Yes |
| Musacchio (2011) | No | Yes | No | Yes | N/A | Yes | N/A | Yes | Yes |
| (2003) | No | No | No | Yes | N/A | No | N/A | Yes | Yes |
| Reuben (2011) | No | N/A | No | Unclear | N/A | N/A | N/A | Unclear | Unclear |
| Sanchez (2011) | N/A | Yes | No | Yes | N/A | Yes | No | Yes | Yes |
| Siminerio (2005) | No | Yes | No | Yes | N/A | Yes | No | Yes | Yes |
| Solberg (2006) | No | Yes | No | No | N/A | Yes | No | Yes | Unclear |
| Wang (2004) | N/A | Not provided | No | Unclear | N/A | Unclear | N/A | Unclear | Unclear |
| Weeramanthri (2012) | N/A | Yes | No | Yes | N/A | Yes | No | Yes | Unclear |
| Wellingham (2003) | Partially | No | No | Unclear | Unclear | Unclear | Unclear | Unclear | Unclear |

# Additional File 7 – Description of Chronic Care Models

| FIRST AUTHOR (YEAR) | Geographical Context | Study Type | CHRONIC Disease Focus |
| --- | --- | --- | --- |
| Barcelo (2010) | Central & South America | RCT | Diabetes |
| Baynouna (2010) | Middle East | Case studies/series | Chronic Diseases Not Specific |
| Boyd (2010) | USA | RCT | Chronic Diseases Not Specific |
| Casas (2006) | Europe | RCT | COPD |
| Chin (2004) | USA | Case studies/series | Diabetes |
| Chin (2007) | USA | RCT | Diabetes |
| Chiou (2001) | Asia | Case studies/series | Diabetes |
| Ciccone (2010) | Europe | Case studies/series | CVD |
| Coleman (1999) | USA | RCT | Chronic Diseases Not Specific |
| Daniel (2004) | USA | Case studies/series | Diabetes |
| Delon (2009) | Canada | Case studies/series | Chronic Diseases Not Specific |
| DiPiero (2008) | USA | Cohort Studies | Diabetes |
| Dorr (2005) | USA | Cohort Studies | Diabetes |
| Dorr (2008) | USA | non RCT | Chronic Diseases Not Specific |
| Esperat (2012) | USA | Case studies/series | Diabetes, Depression |
| Feifer (2001) | USA | Cross Sectional | Diabetes, CVD |
| Friedman (1998) | USA | Case studies/series | Diabetes |
| Fuller (2004) | Australia | Qualitative | Chronic Diseases Not Specific |
| Gabbay (2011) | USA | Case studies/series | Diabetes |
| Glasgow (2002) | USA | Case studies/series | Diabetes, CVD |
| Green (2006) | Canada | Qualitative | Multiple Chronic Diseases |
| Grossman (2008) | USA | Cross Sectional | Diabetes, CVD |
| Olivarius (2001) | Europe | RCT | Diabetes |
| Harvey (2008) | Australia | Case studies/series | Diabetes, CVD, Depression, Respiratory, Renal |
| Hess (2007) | USA | Qualitative | Diabetes |
| Hiss (2007) | USA | RCT | Diabetes |
| Hroscikoski (2006) | USA | Qualitative | Chronic Diseases Not Specific |
| Huckfeldt (2012) | USA | Case studies/series | Diabetes |
| Hung (2008) | USA | Cross Sectional | Diabetes |
| Jackson (2005) | USA | Cross Sectional | Diabetes |
| Jackson (2008) | USA | Cross Sectional | Diabetes |
| Jenkins (2011) | USA | Case studies/series | Diabetes |
| Johnson (2006) | Europe | Qualitative | Diabetes |
| Katz (2009) | Africa | Case studies/series | Diabetes |
| Kimura (2008) | USA | Cohort Studies | Diabetes |
| Landis (2006) | USA | Case studies/series | Diabetes |
| Landis (2007) | USA | RCT | Diabetes, Depression |
| Lemay (2010) | USA | Qualitative | Diabetes |
| Lemmens (2009) | Europe | Case studies/series | COPD |
| Letourneu (2006) | USA | Case studies/series | Diabetes, CVD, Depression |
| Lyon (2011) | USA | Case studies/series | Chronic Diseases Not Specific |
| McCulloch (1998) | USA | Case studies/series | Diabetes |
| McCulloch (2000) | USA | Case studies/series | Diabetes |
| McRae (2008) | Australia | Case studies/series | Diabetes |
| Martin (2012) | Europe | RCT | Chronic Diseases Not Specific |
| Meulepas (2007) | Europe | non RCT | COPD |
| Mohiddin (2006) | Europe | Cohort Studies | Diabetes |
| Montori (2002) | USA | Case studies/series | Diabetes |
| Musacchio (2011) | Europe | Case studies/series | Diabetes |
| Nagykaldi (2003) | USA | Case studies/series | Diabetes |
| Nasmith (2004) | Canada | Qualitative | Diabetes |
| Nundy (2012) | USA | Case studies/series | Diabetes |
| Nutting (2007) | USA | Cross Sectional | Diabetes |
| O’Toole (2010) | USA | Cohort Studies | Chronic Diseases Not Specific |
| Piatt (2006) | USA | RCT | Diabetes |
| Rea (2004) | New Zealand | RCT | COPD |
| Reuben (2011) | USA | Case studies/series | Chronic Diseases Not Specific |
| Rondeau (2009) | Canada | Case studies/series | Diabetes |
| Sanchez (2011) | USA | Case studies/series | Diabetes, CVD, Other Disease Focus |
| Schmittdiel (2008) | USA | Cross Sectional | Chronic Diseases Not Specific |
| Si (2008) | Australia | Cross Sectional | Diabetes |
| Siminerio (2005) | USA | Case studies/series | Diabetes |
| Siminerio (2009) | USA | Case studies/series | Diabetes |
| Smith (2008) | USA | RCT | Diabetes, CVD, Depression, |
| Solberg (2006) | USA | Case studies/series | Diabetes |
| Strickland (2010) | USA | Cross Sectional | Diabetes |
| Sunaert (2010) | Europe | Cohort Studies | Diabetes |
| Sunaert (2009) | Europe | Qualitative | Diabetes |
| Szecsenyi (2008) | Europe | Cross Sectional | Chronic Diseases Not Specific |
| Wagner (1999) | USA | Qualitative | Chronic Diseases Not Specific |
| Walters (2012) | Europe | Qualitative | Diabetes |
| Wang (2004) | USA | Case studies/series | Diabetes |
| Weeramanthri (2003) | Australia | Case studies/series | Diabetes, CVD |
| Weinstein (2011) | USA | Case studies/series | Chronic Diseases Not Specific |
| Wellingham (2002) | New Zealand | Case studies/series | Diabetes, COPD, CVD |
| Wolff (2009) | USA | RCT | Depression |
| Wong (2012) | USA | Cross Sectional | Diabetes |

# Additional File 8 – RCT Study Outcomes

| FIRST AUTHOR (DATE) | FOLLOW UP PERIOD | NUMBER OF PARTICIPANTS | Intervention | OUTCOMES | RESULTS INTERVENTION VS CONTROL |
| --- | --- | --- | --- | --- | --- |
| Barcelo (2010) | 18 months | Intervention: 196  Control: 111 | three learning sessions delivered within 18 months covering general how to care for my diabetes education to patients  above education also provided to primary care personnel teams (including training on foot care and in-service training on diabetes management)  current referral system modified to bring specialists into the practice  advisor provided for case management by visiting intervention clinics | Glycated Haemoglobin (HBA1c) <7% | Intervention OR 1.67 p=0.03 |
|  |  |  |  | Total Cholesterol (TC) <200 mg/dl | Intervention OR 2.31 p<0.01 |
|  |  |  |  | Blood Pressure (BP) | No significant differences |
|  |  |  |  | Diabetes Mellitus processes of care | Foot and eye exam significantly increased compared to control p<0.01 |
| Boyd (2010) | 18 months | Intervention: 485  Control: 419 | comprehensive assessment at home  creation and maintenance of an evidence based “Care Guide” (care plan) and an “Action Plan” (patient’s self-care plan)  monthly monitoring  coaching for self-management  smoothing transitions into and out of hospitals, including coordinating all providers of care  educating and supporting family caregivers  accessing community resources | Patient Assessment of Chronic Illness Care (PACIC) | Quality of care score “high quality” intervention OR 2.13 (1.3-3.5) p=0.003  All PACIC subscales significantly higher except decision support (no significant differences) |
| Casas (2006) | 12 months | Intervention: 65  Control: 90 | comprehensive assessment of patient at discharge and evaluation of social support requirements  educational program on self-management of the disease administered at discharge  agreement on an individually tailored care plan following international guidelines.  accessibility of the specialised nurse patients/carers and primary care professionals during the follow-up period was ensured through an ICT platform including a web-based call centre. | Hospitalisation | Intervention: OR 0.55 (0.35-0.88) p=0.01 |
| Chin (2007) | RCT | Intervention:117  Control: 1190 | Control and intervention: regional QI initiative based around CCM DSD: “Health Disparities Collaborative” team in each centre. ‘Plan-do-study-act’ model introduced; CIS; patient registries to track care.  Intervention only: more intensive support: DSD: learning sessions/workshops to discuss models of care and lessons learnt, clinician-patient communication sessions. SMS: patient empowerment/education materials | Low-density lipoprotein (LDL), HBA1c, blood pressure (BP) | No significant differences |
|  |  |  |  | Diabetes Mellitus processes of care | Intervention: Significantly *less* record of Diabetes Mellitus education and diet and exercise counselling |
|  |  |  |  | Mortality | No significant differences |
| Coleman (1999) | 24 months | Intervention: 5 physicians, 96 patients  Control: 4 physicians, 73 patients | Health system improvement with increased patient visits to a multidisciplinary team that incorporated, CM, SMS and DSD  Also included training in patient management, and more access to patient health information | Geriatric syndromes  Processes of care | No significant differences |
| Olivarius (2001) | 6 years | Intervention: 648  Control: 614 | Intervention: SMS: doctors advised to participate in personalised goal setting and motivation with patients. DS: provision of guidelines to doctors re diabetes management  Control: usual care | HBA1c | Intervention 8.5% *v*  control 9.0%, P<0.0001 |
|  |  |  |  | Systolic BP (SBP) | 145 *v* 150 mm Hg, p=0.0004 |
|  |  |  |  | TC | 6.0 *v* 6.1 mmol/l, p=0.029 |
|  |  |  |  | Diabetes Mellitus related complications, death | No significant differences |
| Martin (2012) | 12 months | Intervention: 153  Control: 61 | Intervention: CM/DS/DSD: lay “care guides” phone patients 1-5 times per week and deliver semi-structured interviews about current health, summaries were entered into “patient journey record system”; computer alerts sent to case manager with recommended action (e.g. visit GP, home visit, go to hospital). Case manager relays alerts to clinician/patient as required.  Control: monthly follow-up and usual care | Hospital admission | 0.051 per person/month vs control 0.1 admissions p<0.052 |
| Piatt (2006) | 12 months | 3 practices in each group:  CCM 30 patients  Provider education 30 patients  Usual care 51 patients | CCM group:  DSD: provider education, diabetes educator employed (6months), GP encouraged to redesign process for routine diabetes visits  SMS: patients encouraged to attend 6 diabetes self-management training sessions (weekly) + support groups (monthly thereafter)  DS: provider education session, guideline material, feedback from audit of medical records  CS: building community partnerships  Provider education group:  DS: education session and results of chart audit discussed only  Usual care group: mailed results of audit without feedback and distributed guideline material. | HBA1c | Intervention: 7.6% (baseline) to >7% (follow up) p=0.008  Control: No significant differences  Significant Between Group Difference p=0.01 |
|  |  |  |  | non-high density lipoprotein (HDL) cholesterol | Intervention: 153.7mg/dl (baseline) to 143.3mg/dl (follow up) p=0.24  Control: No significant differences  Significant Between Group Difference p=0.05 |
|  |  |  |  | SBP and diastolic BP (DBP) | No significant differences |
|  |  |  |  | Diabetes knowledge, empowerment, blood glucose –self monitoring | Intervention: % self-monitoring 77.8% (baseline)100% (follow up)  Control: No significant differences  Significant Between Group Difference p=0.03 |
| Rea (2004) | 12 months | Intervention: 83  Control: 52 | SMS: Patient-specific care plans with set goals, action plan completed  DS: Smoking cessation, inhaler technique, medication review  Flu vaccination  Pulmonary rehabilitation program | Forced expiratory volume (FEV1) | Intervention +2.6% vs control -4.4% (p<0.001) |
|  |  |  |  | Respiratory hospital admissions | Mean bed days intervention -60.7% v control +14.3% p=0.03 for change but between group differences not significant |
|  |  |  |  | Shuttle walk test | No significant differences |
|  |  |  |  | SF-36 | No significant differences |
| Smith (2008) |  | Intervention:  49 physicians, 358 patients  Control: 45 physicians,  277 patients | Tele-medicine intervention consisted of electronic messaging library (specialty advice and evidence-based messages regarding medication management for cardiovascular risk) sent to clinicians.  Control usual care | HbA1c, LDL, triglycerides, SBP and DBP, UK Prospective Diabetes Study 10-year risk, mental SF-36 and Physical SF-36 | No significant differences |
| Wolff (2009) | 6 months | Intervention: 156  Control: 152  [Carer/Patient dyads] | SMS: initial meeting between patients and nurse for 30 mins with education plus ongoing access and coaching by phone and email during business hours, group workshop for family and friends and facilitated once monthly support groups  CS: referral to community resource | Carer depression and strain scores: Center for Epidemiological Studies Depression Caregiver Strain Index | No significant differences |

# Additional File 9 – Non-RCT Outcomes

| First Author (Year) | NUMBER OF PARTICIPANTS | Components | OUTCOMES MEASURED | RESULTS |
| --- | --- | --- | --- | --- |
| Dorr (2008) | Intervention: 1144  Control: 2288 | Intervention:  CM: Case manager provides education, motivation, follow-up, referral to community resources  CIS/DS: enhanced clinical info system for intervention and control with electronic medical record and guidelines/chronic disease reminders. Case managers have additional alerting system. This portion of intervention relates only to the care manager’s use of the technology  Control: usual care + enhanced CIS as above. | Mortality and hospitalisation | Intervention vs Control (all patients):  Deaths at 1 year: OR: 0.68 (p<0.05)  Emergency department (ED) visits at 2 years: OR: 1.28 (p<0.05)  No significant differences for deaths at 2 years, ED visits at 1 year, all hospitalisations at 1 and 2 years and hospitalisations at 1 and 2 years.  Intervention vs Control (diabetes patients):  Deaths at 1 year: OR: 0.56 (p<0.05)  Non-significant findings for all other measures 1 and 2 years. |
| Meulepas (2007) | Intervention: 22 practices, 137 patients  Control: 22 practices, 123 patients | Intervention:  DSD/CIS: integrated care provided by: GP, practice nurse, logistic COPD support service, chest physician. Use of patient register and recall system  SMS: practise nurse addresses inhalation technique, counselling, smoking cessation  Control: usual care | Successful delegation of tasks from GPs to Chronic Obstructive Pulmonary Disease Support Service and Practice Nurse  Performance in daily practice according to model components. | All practices introduced:  Patient register and recall system (100% led by practice nurse)  Periodical case history reporting and lung function measurements (59% Practice Nurse)  Periodic visit arrangements (68% Practice Nurse led)  Provision of advice and counseling to protocol (100% Practice Nurse led) |

# Additional File 10 – Retrospective Study Outcomes

| FIRST AUTHOR (YEAR) | FOLLOW UP PERIOD | NUMBER OF PARTICIPANTS | DISEASE | OUTCOMES | RESULTS INTERVENTION VS CONTROL |
| --- | --- | --- | --- | --- | --- |
| Dipiero (2008) | 6-24 months | Exposure: 288  Control: 277 | Diabetes mellitus | HBA1c <7% | OR: 1.7 (1.02-3.0) |
|  |  |  |  | BP<130/80 | OR: 2.8 (2.1-4.5) |
|  |  |  |  | LDL | No significant differences |
|  |  |  |  | Process measures | Significant improvements in annual testing of LDL, micro-albumin, BP, long term complications |
| Dorr (2005) | 12 months | Exposure: 1185  Control: 4740 | Diabetes mellitus | HBA1c<7% | OR: 1.31 (1.14-1.51) p<0.01 |
|  |  |  |  | LDL | NSD |
|  |  |  |  | HBA1c testing overdue | OR: 0.79 (0.71-0.85) p<0.01 |
| Kimura (2008) | 3 years | Exposure: 2809  Control: 5688 | Diabetes mellitus | HBA1c, LDL, BP testing  HBA1c, SBP, LDL control | No statistical testing performed, trend toward better process measures in control, limited clinical marker differences |
| Mohiddin (2006) | 3 years | Exposure: 1360 (17 practices)  Control: 739 (9 practices) | Diabetes mellitus | HBA1c annual testing | Both groups: significant increase over time no between-group differences |
|  |  |  |  | Cholesterol annual testing | Both groups: significant increase over time no between-group differences |
|  |  |  |  | BP annual testing | Both groups: significant increase over time, control practices significantly greater increase. |
| O’Toole (2010) | 12 months | Exposure: 79  Control: 98 | Chronic disease (non-specific) | BP <140/90 | No significant differences |
|  |  |  |  | LDL target | 65.4% (exposure) vs. 45.5% (control) p<0.01 |
|  |  |  |  | ED visit total/ non-acute | No significant differences.  Exposure group: significantly lower odds of visit for non-acute condition OR: 0.4 (0.2-0.8) |
|  |  |  |  | Hospital admission total/ by presenting complaint | Control reduced hospital admission significantly compared to intervention/ proportion not related to drug/alcohol/mental health fell significantly in exposure (28.6% 🡪10.8%) compared to control (p<0.01). |
| Sunaert (2010) | 3 years | Exposure: 2425  Control: 1749 | Diabetes mellitus | HBA1c<7% | No significant differences |
|  |  |  |  | TC<190mg/dl (proportion) | +18.32% (exposure) vs +12.8% (control) p=0.03 |
|  |  |  |  | HBA1c annual test  TC annual test  Statin therapy | +5.73% (exposure) vs +2.02% (control) p=0.01  +0.8% (exposure) vs -2.59% (control) p=0.02  +18.08% (exposure) vs +12.39% (control) p=0.0002 |
|  |  |  |  | Microalbumin test  Opthalmologist visit | No significant differences (very low rates, <30% both groups)  No significant differences (<40% both groups) |

# Additional File 11 – Cross Sectional Study Outcomes

| FIRST AUTHOR (YEAR) | NUMBER OF PARTICIPANTS | Implementation Strategies | OUTCOMES measured | RESULTS |
| --- | --- | --- | --- | --- |
| Feifer (2001) | n=9 practice sites #patients not specified | No intervention | Relationship of provider reported Assessment of Chronic Illness Care (ACIC) CCM elements against clinical outcomes. | Clinical outcomes:  Summary ACIC score positively correlated with the best practice index (r=0.828, p=0.006)  CCM elements correlated with best practice performance:  DS: r=0.907 (p=0.001)  SMS: r=0.845 (p=0.004)  DSD: r=0.803 (p=0.009)  CCM element activities correlated with clinical outcomes:  Integrated guidelines: r=0.688 (p=0.04)  Care team leadership: r=0.816 (p=0.007)  Follow-up: r=0.744 (p=0.02)  Self-care education and support: r=0.761 (p=0.02)  Behavioral therapy: r=0.694 (p=0.04)  Process outcomes:  Lowest average ACIC item score for specialist involvement in provider decision making. |
| Grossman (2008) | 44 health centres | Study investigated how QI initiatives supported CCM elements | Exploratory analyses: association of CCM elements with quality of care improvements. | No significant relationship between measures of QI intensity and changes in the observed quality of care (all p>0.05)  Process outcomes:  53% of CCM activities institutionalised and/or refined  28% CCM activities evaluated  SMS and DSD most likely to be institutionalised and/or refined.  50 (3%) activities rated as high or very high probable impact on quality. These activities included collaborative decision making with patients or institutionalisation of guidelines, protocols and prompts. |
| Hung (2008) | n=57 practices  n=4735 patients | No intervention | Relationship between CCM and patient health measures: general health status and health-related quality of life (HRQOL). | General health status:  Lower health status reported for patients using:  Hospital/university health system–owned practices (OR: 0.73, p<0.01)  Multispecialty practices (OR: 0.67, p<0.01)  group/individual planned visits for prevention (OR: 0.81, p<0.05)  Personal Digital Assistants for clinician-decision support (OR: 0.61, p<0.01)  Better health status reported for patients in practices that supported:  Behaviour change (OR: 1.98, p<0.05)  Health promotion champions onsite (OR: 1.47, p<0.001)  patient registries (OR: 1.33, p<0.01)  Quality of Life:  Higher number of unhealthy days reported with practices using group /individual planned visits for prevention and patient reminder cards (OR: 0.69, p<0.001)  Lower number of unhealthy days reported with practices using:  Patient registries, health promotion champions and evidence-based guidelines (OR: 1.29-1.41, p<0.01)  Public reporting of performance measures (OR: 1.28, p<0.01)  More electronic medical record functions (OR: 1.08, p<0.01). |
| Jackson (2005) | Total 224,221 patients, 82,428 patients, with mean HbA1c of 7.6% | No intervention | HBA1c | Lower HBA1c (p<0.1)associated with:  Greater authority to establish or implement clinical policies  Greater staffing authority  Greater use of gatekeeping (maintaining level of control over referrals)  Computerised diabetes reminders  Special teams or protocols to respond to clinical issues  Multidisciplinary weekly clinical team meetings  All physicians (but not all nurses) involved with QI  Notifying all patients of assigned provider  Hiring needed new staff  Having nurses that report only to the program  Being a large academic practice.  Higher HBA1c (p<0.1)associated with:  Programs reporting that patients  almost always see their assigned provider  Having a QI program involving all  nurses without all physicians  Having general internal medicine physicians reporting only to the program  Being located at an acute care hospital  Having a greater number of reported providers. |
| Jackson (2008) | 186 patients | No intervention | Level of consistency between Patient Assessment Chronic Illness Care (PACIC) experiences with the diabetes care system and CCM | Compered to White patients, non-White patients reported higher levels of:  Assistance with problem solving OR: 1.57 (1.14–2.16)  Follow up care OR: 2.86 (1.41–5.82)  Compared to patients with higher levels of education, patients with lower levels of education reported higher levels of:  Patient activation OR: 2.12 (1.00–4.50)  Delivery system design OR: 3.77 (1.28–11.08)]  Goal setting OR: 3.21 (1.34–7.69)  Problem solving OR: 3.11 (1.11–8.71) |
| Nutting (2007) | 90 clinicians  886 patients | No intervention | Clinician reported:  Use of CCM elements  HBA1c  Lipid levels | Clinician reported:  Use of CCM elements significantly associated with:  Lower HbA1c: parameter estimate=-0.3013 (p=0.002)  Lower lipid profiles (TC:HDL ratio): parameter estimate= -0.1663 (p=0.02)  Patient reported use of CCM elements significantly associated with  Better behavioral care: parameter estimate=0.2105 (p=0.001)  Better clinical care: parameter estimate=0.1592 (p=0.07) |
| Schmittdiel (2007) | n=4,108 | No intervention | Association between PACIC score and:  self-management resources  adherence to medications  quality of life | The PACIC was significantly and positively associated with:  Use of at least one self-management service OR: 1.40 (1.30, 1.53)  Greater engagement in all self-management behaviours OR: range 1.21 to 1.41)  Higher rating of health care OR: 2.36 (2.18, 2.56)  Higher quality of life OR: 1.20 (1.13, 1.27) |
| Si (2008) | 12 health centres | No intervention | Extent to which Indigenous community health centres support chronic illness care | No direct reporting of CCM element efficacy or correlation with level of implementation. |
| Strickland (2010) | 25 practices  674 patients | No intervention | Association between CCM implementation and:  diabetes care  diabetes counselling | Comprehensive CCM implementation associated with increased:  Assessment of HbA1c, lipids and blood pressure OR: 1.90 (p=0.009)  Treatment for HbA1c, lipids and blood pressure OR: 1.79 (p=0.015)  Physical activity counseling for obese and overweight patients OR: 1.35 (p=0.0017).  Practices more “open to innovation” were more lively to offer physical activity counseling to overweight and obese patients OR: 1.60 (p=0.0006). |
| Szecsenyi (2008) | 1,399 patients | No intervention | Differences in care experienced by patients as measured by PACIC. | Compared to patients who did not receive disease management program, patients who did reported receiving better care:  Patient activation (p<0.05)  Delivery system/practice design (p<0.001)  Goal setting/tailoring (p<0.001)  Problem solving (p<0.001)  Follow up coordination (p<0.001) |
| Wong (2013) | 28 physician organisations  2162 diabetic patients | No intervention | Association between care management processes, integration of  services, and BP control | Significant correlations between:  Service integration and BP control (<130/80) r=0.38 (p=0.05)  Structure integration and BP control  r=0.49 (p=0.01)  Clinical info systems BP control (>140/85) r=-0.38 (p=0.04) |

# Additional File 12 – Case Study and Case Series Outcomes

| Paper | NUMBER OF PARTICIPANTS | Implementation Strategies | OUTCOMES measured | RESULTS |
| --- | --- | --- | --- | --- |
| Baynouna (2010) | Audit of clinic records  2004 – 672 files audited  2005 – 164 files audited  2007 – 1402 files audited  2008 – 715 files audited | Used a Quality Assurance cycle with community survey and an audit of the health care system undertaken emphasising chronic disease care. Feedback provided to staff, who then implemented a Plan-Do-Study- Act method to identify deficiencies in health care and develop interventions  or improvements. | Outcomes focused on diabetes management including documentation of patient information. Also measured body mass index (BMI), smoking status, HbA1c in diabetic patients, SBP and DBP in diabetics, LDL, triglycerides and HDL, medication and ophthalmology referrals | Documentation of patients history, examinations and investigators showed significant improvements including recording of BMI  BP outcomes improved significantly with around two thirds of patients meeting target values of 140/90 or less in 2007 and 2008  HbA1c in diabetics dropped significantly with 80% having values below 9 in 2008 compared with 61.7% in 2004 and 29.5 in 2006.  LDL, total cholesterol and triglycerides significantly improved with more than three quarters of patients meeting target values in 2007 and 2008 compared to only 1 third in 2004 and half in 2006  Changes in disease control measures reflected significant increase in prescriptions of aspirin & statins  Ophthalmology referrals markedly increased from 5.5% in 2004 to 50.8% in 2007 but decreased to 31.3% in 2008  Referral, dietary counseling, foot examination, dental referral, lipid assessment and urine micro albumin assessment all improved with scores ranging from 1.28 to 2.92 |
| Chin (2004) | 19 primary care services  106 healthcare providers  1520 patient records audited at two time points | Diabetes quality improvement model including rapid Plan-Do-Study-Act cycles. | Chart Reviews focused on process of care measures based on clinical recommendations | The proportions of patients for whom each of the major processes of care were performed increased significantly from 1998 to 1999 |
| Chiou (2001) | 696 charts audited | Lan-Yang Diabetes Shared Care System including regional guidelines for diabetes management, multidisciplinary diabetes care teams organised and provided with training and certification process. Physicians used shared referral protocols and sheets. | The proportions of patients receiving at least one measurement of HbA1c, urine micro-albumin or fundus examinations and means of HbA1c during the period before, during and after the implementation of the system. | The mean proportion of diabetics monitored for glycemic control, renal and retinal conditions significantly increased with the establishment of the shared care system. |
| Ciccone (2010) | 20 GP group offices  83 physicians  30 care managers  1,160 patients | Collaborative team consisting of physicians, care managers, specialists,  and patients. Guidelines and recommendations for each CD condition developed in order to improve patient health outcomes  and promote appropriate resource utilization. | SF-12, Morisky Compliance Scale, blood pressure (BP, cholesterol glycosylated haemoglobin blood level, level of routine assessment and monitoring of blood pressure at home visits, self-monitoring of blood glucose, monitoring of symptoms for heart failure (HF) | 70% of patients received recommended tests and services as appropriate  Statistically significant increase in the number of days per week employed for physical training (p<0.0001) increase in time spent doing physical activity from 19.87 to 32.90 minutes (p< 0.001) per session  Statistically significant change in BMI, LDL, SBP and total cholesterol  Significant decrease in both DBP values (p<0.001) from initial to final measurement  Average SF-12 score 7.99 above the nation normal value and 5.28 above the starting score of the population studied |
| Daniel (2004) | Diabetes Collaborative I comprised of:  3 government or public health care delivery systems,  3 small community care clinics,  4 large clinics,  4 hospital systems  3 private medical practices  Diabetes Collaborative II comprised of 30 teams and 6 health plans. | Learning collaboratives consisting of a number of healthcare teams designed to support implementation. | **Process Measures**  HbA1c measure in past 12 months  BP measurement in past 12 months  LDL cholesterol test in past 12 months  Food examination in past 12 months  Referral for retinal examination in past 12 months  Documented SM goal in past 12 months  **Outcome measures**  Most recent HbA1c <8.0% among those receiving a test in past 12 months  Most recent BP ≤ 140/90 mm Hg among those tested in past 12 months  Most recent LDL cholesterol < 130 gm/dl among those receiving a test in past 12 months | ABSOLUTE Improvement, % for:  Process Measures for Collaborative I  HbA1c = 27  BP = 29  LDL = 35  Foot exam = 50  Referral for retinal exam = 30  Documented SM Goal =31  Process Measures for Collaborative II  HbA1c = 6  BP = 2  LDL = 24  Foot exam = 24  Referral for retinal exam = 7  Documented SM Goal =34  Outcome Measures for Collaborative I  HbA1c = 12  BP = 2  LDL = 13  Outcome Measures for Collaborative II  HbA1c = 7  BP = 7  LDL = 2 |
| Delon (2009) | Not provided | Outcomes from Alberta’s CDM program described. | Not specified | 17% increase in the percentage of diabetic patients with A1c control (from 40% to 56% between baseline and one year follow-up  13% increase in the percentage of dyslipidemia patients with triglyceride control (from 34% to 47%) between baseline and one-year follow-up  19% decrease in-patients with a COPD-related exacerbation resulting in an inpatient hospitalization (from 320 to 260 per 1,000 patients) between baseline and one-year follow-up  41% decrease in in-patient hospital admissions across all patients (from 380 to 224 per 1000 patients) between baseline and one-year follow-up  34% decrease in emergency department visits across all patients (from 755 to 495 per 1,000 patients) between baseline and one year follow-up |
| Esperate (2012) | 152 patients | Transformation Para Salud Program utilising Community Health Workers (CHWs) to provide information to their patients about effective use of community resources to address their sociocultural needs.  A network of referral systems was formalized to aid CHWs to facilitate patient’s abilities to face demands that social determinants of health imposed upon them. | Clinical indicators targeted for each disease process:  SBP and DBP  HbA1c  Cholesterol, triglycerides, LDL and HDL  ED visits  BMI  Depression PHQ-9  Diabetes self-efficacy, chronic disease self-efficacy, personal resource questionnaire. | At the end of the two year demonstration project statistically significant improvements to average HbA1c ( 9.1 to 8.4) and BP.  Compared to baseline, the score for self-efficacy of chronic disease management and diabetes self-efficacy after navigation program had both significantly improved 1.07 (p<0.001) and 1.13 (p< 0.001).  Diabetes clients showed increased days per week for following a healthful diet plan (M=0.68, SD=2.05, p<0.05), having five or more servings of fruits and vegetables per day but less high fat foods (M=0.51, SD=1.78, p<0.05), doing more exercise (M=0.79, SD-2.34, p<0.05), following doctors’ recommendations on blood sugar testing (M=1.01, SD=2.81, p<0.05), and checking the feet(M=1.37, SD2.61, p<0.001). |
| Friedman (1998) | 1457 patients | Multidisciplinary EPISODES OF CARE team implemented program which included Physician-Provider Interventions  Practice guidelines  Medical profile screens  Diabetes provider support report  Patient interventions  Focused diabetes clinic visits  Patient education  Diabetes days  Reminder systems | Glyco-hemoglobin values  Dilated eye examination rates  Educational access rates | Mean glycol-hemoglobin values decreased from 12.2% ±3.09% in 1994 to 11.39%±2.92% in 1995 (p<0.01) and 10.4%±2.66% in 1996 (p<0.005)  Examination rates for participants have improved from 47.3% in 1994 to 52.6% in 1995 and 53.2% in 1996  Of the participants receiving reminder letters, 17% to 20% subsequently had dilated eye examinations  % of participants seen by the diabetes educators has increased from 52% in 1993 to 78% in 1995. |
| Gabbay (2011) | 25 primary care practices  143 health providers  Patients n=10,016 | Plan-Do-Study-Act learning collaborative developed to support the implementation of the CCM and a Patient-Centered Medical Home which includes a physician led team of individuals who have an ongoing relationship with the patient and when appropriate their family | A1c1  Blood pressure  Dilated eye examination  Foot examination  Nephropathy  Tobacco use  Influenza vaccination  Evidence based treatments  Precent of all diabetes patients meeting the indicated parameters | 12 practices achieved recognition under the National Committee for Quality Assurance Physician Practice Connections Patient-Centered Medical Home Recognition  There was significant improvement in both evidence-based care guideline adherence and in clinical outcomes:  % of patients who received a yearly foot assessment for neuropathy increased significantly from 50-69%  % of patients receiving yearly screenings for nephropathy and diabetic retinopathy as well as administration of pneumonia and influenza vaccines also improved  Use of therapies improved significantly:  Angiotensin-converting enzyme inhibitors  Angiotensin receptor blocking agents  Statins  ACEIs or ARBs  Evidence based aspirin  Diabetes measures showed a small statistically significant improvement:  8.5% absolute increase in the percentage of patients with LDLc <130  4% absolute increase in the percentage of patients with BP < 140/90  2.5% absolute decrease in the percentage of patients with A1C >9  More patients achieved the LDL target of < 100 |
| Glasgow (2002) | 21 health care teams  14 HF  7 diabetes | Plan-Do-Study-Act cycle to implement SMS and HS, as well as develop and refine quality improvement plans. | All teams chose three to five outcome measures related to their goals for quality improvement. Teams submitted data on these measures throughout implementation. In addition, self-ratings of their self-management support were captured. | The median levels for daily self-monitoring of weight by patients improved dramatically from 19% at baseline to 93% at the end of the Collaborative. The only other team goal on which complete data were available for HF was providing instructions in self-management which improved from 68% at baseline to 90% at the end of the collaborative.  Three of the seven diabetes teams submitted SMS data and these results revealed consistent but less dramatic improvement than shown by HF teams. All teams improved at least 10%.  93% of HF teams and 71% of diabetes teams rated themselves as having average or slightly below average SMS capabilities at the beginning of the collaborative. Significant SMS score improvement was seen for HF teams at the end of the collaborative, with teams reporting an average improvement of 3.2points. Although scores also improved for diabetes teams there were not significant. |
| Harvey (2008) | 175 patients | The Sharing Health Care  offered patients a range of education and  support options including a 6-week peer-led CD self-management program. | Modified Stanford 2000 Health Assessment self-report survey  Patient and Clinician Partners in self-rated health questionnaire administered at baseline and then again at 6, 12 and 18 months post intervention | Stanford 2000 Health Survey showed reductions in:  Specifically health service utilisation (number of visits to GPs, specialists and hospitals),  Impact of pain, worry about illness  Frustration with illness and fear of the future  Partners in Health Self Rated Questionnaire:  The majority of scores improved with time  For both patients and providers showed statistically significant improvements being made in all domains apart from Question 3.  Statistically significant improvement in patient self-management knowledge and skill |
| Huckfeldt (2012) | Group one: Convenience sample of 244 records for diabetic patient records in two Queens Care Family Clinics  Group two: Comparison Group records for 1622 diabetic patient records in a Disease Management Program short-term intensive intervention | Description of two intervention models:  Model one: interaction of case management and clinical pharmacy (education) program into primary care in a community clinic  Model two: a sort-term intensive intervention by a care team including nurses and a specialist | Provided following measures for both groups but did not specifically undertake a comparison:  HbA1c arrange  LDL | Group One:  Records for case management approach showed a significant improvement (p<0.001) in HbA1c and LDL cholesterol.  Records for clinical pharmacy (education approach) showed a significant improvement (p<0.001) in HbA1c and LDL Cholesterol  Group Two:  Records from the Disease Management Program at six department of health services clinics should unadjusted decreases in both HbA1c and LDL  Both models improved short-term disease control |
| Jenkins (2011) | Total population of Georgetown and Charleston who have been diagnosed with diabetes (no. not provided) | Described the Community Chronic Care  Conceptual Model for REACH Charleston and  Georgetown Diabetes Coalition which focused on:  Evidence-based health systems using continuous quality improvement  Community-driven educational activities  Creation of healthy learning  Coalition power to foster collaboration, trust, and sound business planning | Chart review:  Foot examinations conducted by REACH health care system partners  HbA1c tests completed  Lipid tests completed  Rates of amputations | Foot examinations conducted by REACH healthcare system partners increased significantly for both genders of African and white Americans with diabetes  Annual foot examinations for all patients with diabetes were more than 90% for African American males and females and more than 85% for whites in 2008  Increases in % of African American patients with diabetes receiving an annual HbA1c test increased from 76.8% to more than 97%  Increases in annual lipid testing increased from 47% to almost 90%  Rates of amputations per 1000 diabetes hospitalizations decreased significantly  Rates of amputations per 100000 population decreased significantly |
| Katz (2009) | 257 patients  186 primary healthcare nurses | All clinics were incorporated into a program modelled  on the CCM which utilized primary health care nurses (PHCNs) to link primary care and specialist care. PHCNs  were provided with decision support, escalated scaling up of  medication and prompt access to specialist care. | The number of patients correctly enrolled to the Chronic Disease Outreach Program and referred to specialists | Sensitivity for primary healthcare nurses detecting those patients needing referral was 95% and specificity for those not requiring referral was 100% |
| Landis (2006) | 6 health services.  1,203 diabetic patients at baseline  1,262 patients from five sites at follow-up | Developed a learning collaborative with family  medicine residency sites using the CCM to improve diabetes care according to the National Committee for Quality Assurance and the American Diabetic Association Physician  Recognition Program criteria | The National Committee for Quality Assurance in conjunction with the American Diabetic Association Physician Recognition Program target for diabetes not previously met | Four of the six sites met or exceeded at least one Physician Recognition Program target for diabetes care. Three of the six sites met or exceeded two diabetic targets. One site improved enough to be eligible for the Recognition Award. |
| Lemmens (2009) | 54 healthcare practitioners | Evaluation of physicians commitment to the COPD Management Programme which consisted of patient education, protocoled assessment and treatment of COPD,  and coordination of care. Practice nurses systematically educated  patients on smoking behaviour, medication usage, nutrition and physical activity.  Professionals were educated on the guidelines and programme. | Professional commitment  Organizational context  Programme implementation  Professional commitment to changing chronic illness care | Comparing baseline and post-intervention scores showed that professional commitment (p=0.02)) and valence (the attractiveness assigned to the outcomes associated with successful implementation) ((p=0.009) had improved significantly after the intervention  After the intervention positive collegial response to tasks and behaviours improved significantly (p<0.001).  Extent to which professionals felt that the disease management program was actually implemented improved significantly after intervention  Changes in self-reported SMS and actual application of patient education in practice correlated significantly  Changes in DSD correlated significantly with provision of inhalation instruction  Changes in DSD correlated significantly with the proportions of patients regularly followed up  All showed that all patients underwent spirometry as indicated by guidelines, validating reported improvements to DS |
| Letourneau (2006) | Records from various primary, ambulatory and hospital care services which are part of the MaineHealth Group – Numbers not clear | Learning Collaboratives – Rapid-cycle change used. Teams brought together for a learning sessions. Team then identified its population and target condition to initiate system changes and track progress. | Asthma  Key asthma measures including increased documentation of asthma severity classification and use of controller medications in patients with persistent asthma  Cardiovascular Disease  Left ventricular ejection fraction  ACE inhibitor or ARB for left ventricular systolic dysfunction  Complete HF discharge instructions  Tobacco status addressed  31 day readmission rate for HF  Diabetes  HbA1c  BP  LDL  Depression  PHQ-9 | Asthma  Practices participating in the asthma learning collaborative made significant improvements (p < 0.05) on key asthma measures  Practices using the CIR also demonstrated significant improvements in all asthma measures from December 2004 to December 2005  Cardiovascular Disease  Hospitals and providers made significant improvements (p<0.05) from 2001 to 2005 in all targeted areas  31 day readmission rate for HF in participating hospitals decreased from 17.5% to 7.2%  Diabetes  Participating practices demonstrated significant improvements in both process and outcomes of care (p< 0.001 for improvement in outcomes tracked)  Depression  Fifty primary care clinicians were trained over a 6 month period.  Increase in number of people with diabetes who were screened for depression. |
| Lyon (2011) | Healthcare provider in one practice and their patients | Began by seeking buy-in from staff and formed a leadership team to educate and motivate other providers. Provided training on motivational interviewing, stages of change theory, goal setting, action plan development and patient self-management support. Then formed multidisciplinary teams to continue to improve the system using the plan-do-study-act method to overcome any barriers to implementation. | % patients with A1c <8  % patients with A1c checked in last 12 months  % patients with Lipids checked in past 12 months  Up to date pneumonia vaccination | A1c < 8 – 35% at baseline 52% at most recent measure  A1c checked in 12 mths - 65% at baseline 80% at most recent measure  Lipids checked in 12 mths – 53% at baseline 71% at most recent measure  Up to date pneumonia vaccination – 61% at baseline 85% at most recent measurement |
| McCulloch (1998) | 25 primary care clinics with 200 primary care providers  Comparison of outcomes data:  30 primary care providers that made joint visits with the diabetes expert team and had at least 20 diabetes patients  30 primary care providers that had not made joint visits with the diabetes expert team  Provider satisfaction:  80 randomly selected providers in 1992 and then 60 in 1995. | Developed program based on:  a continually updated on-line registry  evidence-based guidelines  improved support for patient self-management;  practice redesign to encourage group visits; and  decentralized expertise through a diabetes expert care team. | Monitoring and risk factors improvements overall  HbA1c testing  Dilated retinal examination  Foot evaluations  Urinary micoalbumin tests  Prevalence of smoking  Monitoring and risk factors improvements comparison for diabetes expert team  Rates of dilated retinal examinations  % of patients with at least one glycol-haemoglobin test | Monitoring and risk factors  Prevalence of HbA1c testing was approximately 77% increased to 80% by 1996  Prevalence of retinal screen was 46% in 1993 to ~67% in 1996  Foot evaluation 50% of all diabetic patients had a recorded foot examination by June 1997. Prior to 96 suspected ~20% although not recorded  Microalbuminuiria tests increased from under 100 up to April 1996 to 800 by October 1996 after guideline implemented  The prevalence of smoking decreased from 14% in 1994 to 10% in 1996  Monitoring and risk factors improvements comparison for diabetes expert team  Rates of dilated retinal examinations among group A increased from 52.4% in 1994 to 57.5% in 1996 whereas rates in group B were unchanged (p<0.001)  % of patients with at least one glycol-haemoglobin test rose from 78.5% in 1994 to 85.8% in 1996 among group A, were as rates in group B remained unchanged (p<0.03) |
| McCulloch (2000) | Routinely collected data ~15,000 patient records  Questionnaire on patient satisfaction ~1,000 per year  Other indicators an audit of 275 charts | Evaluated program based on:  a continually updated on-line registry  evidence-based guidelines  improved support for patient self-management;  practice redesign to encourage group visits; and  decentralized expertise through a diabetes expert care team. | Dilated retinal examinations  Foot Assessment  Microalbuminuria Screening  Glycaemic control | For all four measures, there has been a steady increase in guideline compliance  Entered on the registry from 18% of patients in 1996 to 80% of patients in 1998  % of patients with at least on HbA1c test in previous year rose from 75% in 1995 to over 90% in 1998  HbA1c for the entire population of patients with diabetes dropped below 8.0% in 1996 and is currently 7.58% |
| McRae (2008) | 74 patients who registered at type 2 diabetes diagnosis and completed information approximately 5 years after diagnosis  6 patients who registered at type 2 diabetes diagnosis but had deceased within the following five years | Description of a program which facilitates GP based case management  by provision of information and education to the  GPs and by direct service provision to patients. The program funds diabetes education  programs, dietician services and an exercise program, and arranges access to podiatry services. | Discounted life expectancy and quality adjusted life expectancy (QALE) | The program is estimated to achieve:  an increase in discounted life expectancy of 9.36 years  an increase in discounted QALE of 0.30 years. |
| Montori (2002) | 3 sites planned care intervention  2 of these sites also implemented a Diabetes Electronic Management System  200 randomly selected patients from each site | Planned care program utilising practice guidelines, SMS and CI. Guideline implementation team comprising of diabetes nurse educator, physician leader and local key personnel. Each team determined goals and designed site-specific strategies. CI systems including feedback to providers implemented. | Standard performance measures during the 12 months before each of the two index visits  Clinical process measures  Laboratory measures  Counselling PRP weighted criterion score to control for multiple clinical testing  Metabolic outcomes  10 year coronary risk using the Framingham coronary risk prediction score  Number of physician, emergency room, and hospital visits | Planned care had a differential impact on performance measures after 2 years of implementation associated with improvement in measurement of:  HbA1c (OR 7.0[95% CI 4.2-11.6])  HDL cholesterol (5.6 [4.1-7.5])  Microalbuminuria [5.3[3.5-8.0])  Provision of tobacco advice(6.9[4.7-10.1])  Improved performance in the ADA PRP weighted criterion score (p=0.0001)  Planned care was associated with significant deterioration in documentation of self-management supports  Diabetes Electronic Management System (DEMS) use had a positive impact on all performance measurements and was associated with a greater improvement than that seen with planned care. DEM was associated with significantly improved frequency of measurement of:  microalbuminuria (OR 3.2[95% CI 1.9-5.2])  documentation of retina examination (2.4[1.5-3.9])  foot examinations (2.3[1.2-4.4]),  self-management support (2.6[1.7-3.8])  diet (1.9[1.2-3.0])  exercise advice (2.7[1.6-4.5]).  Hba1c (4.5[1.0-19.5])  TC (1.4[0.8-2.3]) (not significant)  DEMS high intensity use was associated with significantly greater compliance with the measurement of:  Microalbuminuria (4.0 vs. 60%, p<0.001)  TC, HDL and triglycerides (65 vs 100% p<0.001)  Documentation of SMS (44 vs 81%, p<0.001) |
| Musacchio (2011) | Clinical records of 1004 patients | The implementation of SINERGIA, a model based on a process of disease  monitoring and management that excludes specialist  intervention in the absence of acute  problems. It involves:  Training of the team to identify appropriate patient care  Comprehensive assessment at 1^st^ visit  Other staff take charge if appropriate after 1^st^ visit  Patient empowered to manage own condition | Favourable intermediate outcome indicators:  % of patients with levels of HbA1c ≤ 7.0%  BP ≤130/85 mmHg  LDL cholesterol , 100 mg/dl  Unfavourable intermediate outcome indicators:  Levels of HbA1c ≥ 9%  BP ≥140/90 mmHg  LDL cholesterol ≥ 130mg/dl | The rate of patients at HbA1c target was significantly increased from 32.7 to 45.8%  Positive impact on levels of LDL cholesterol from 39.7 to 47.3%  Mean number of visits per patient/year from 2.8 to 2.3 equating to 500 appointments were avoided in year 1  % of patients treated with insulin remained substantially unchanged  % of patients treated with two or more anti-hypertensive agents changed from 37.1 to 37.8% during follow-up,  proportion of patients treated with lipid-lowering drugs increased from 46.5 to 48.4% |
| Nagykaldi (2003) | 20 family physicians | Describes the development and testing of a personal digital assistant-based diabetes management system and diabetes Toolkit | Measurements for:  HbA1c  Urine for protein  Lipid panel  Retinal examination  Foot examination  Use of angiotensin converting enzyme inhibitor for BP and proteinuria  Flu shot  pneumovax | significant improvement in nine of 10 measures included in the audit (p<0.05)  absolute increase in these values was between 3% and 25%  The short study period probably made documentation of eye exams and impacted on flu-shot rates  Strong correlation with the quality indicator improvement of the measurement and documentation of HbA1c values measured in the last 3 months (p<0.005)  Practice Enhancement Assistants visits increased the number of foot exams and retinal exams performed in the last year (p=0.03 and 0.02) |
| Reuben (2011) | 43,000 (total number of CareMore members a health insurer) | The CareMore model is team based, evidence based, information technology based model. Focused on seniors, it involves:  1-hour Healthy Start visit at a Care-  point-of-care laboratory testing; an inventory of diseases  screening for conditions such as dementia, depression, and falls; medications  Based on the findings of these visits, members are triaged to chronic disease care management programs | Glycoslated haemoglobin for seniors with diabetes  Amputation rate for seniors with diabetes  Hospitalizations for end-stage renal disease  30 day rehospitalisation rates compared with Medicare population  Average length of hospital stay  Annual Consumer Assessment of Healthcare Providers and Systems score | Average of glycosylated haemoglobin level of 7.08  Amputation rate is 78% less than the national average  Hospitalizations for end stage renal disease are 42% less than national average  30 day re-hospitalization rates are 13.6% compared with 20% in the overall Medicare population  Average length of hospital stay is 3 days |
| Sanchez (2011) | 70 patients at baseline, 65 at 2^nd^ visit and 49 at 3^rd^ visit | A quality improvement project using the Plan-  Do-Check-Act cycle was implemented in a primary care setting to provide diabetes self-management  education for adults | Blood pressure  A1C  Cholesterol  Triglycerides  LDL  Urine Albumin | The % of patients maintain BP ≤130/80 between 2^nd^ and 3^rd^ visits were 82% and between 1^st^ and third visits 2343 76%.  There was no statistical change in A1C value from baseline to second and third measures  There was no statistical change for triglycerides from baseline to 2^nd^ visit and between 1^st^ and 3^rd^ visit.  There was no statistically significant change in LDL values between baseline and 2^nd^ visit. |
| Siminerio (2005) | 6 healthcare providers  104 patients  11 patients who also received self-management education | Describes how certified diabetes  educator educated and supported providers on diabetes management and adherence to the  American Diabetes Association (ADA) Standards of  Care. They also provided  diabetes self- management education. | Diabetes Attitude Scale  Diabetes Empowerment Scale  Diabetes Knowledge Test  Diabetes Self-management Program of Health System Initial Assessment | Process measures for all patients:  Significant improvement in adherence to American Diabetes Association Standards of Care  Clinical values exceeding therapeutic goals set forth by the ADA declined but not significantly  Patients who also received self-management education:  significant improvement in mean A1c from baseline (7.2% vsw 6.5% p= .007)  Change in percentage of subjects with an A1c (≥7% (52.9% vs 38.5%, P=.32)  Improvement in HDLc levels (40.5 mg/dL vs 44.4 mg/dL, p=.05)  Mean LDLc levels significantly worsened over the 1 year intervention (85.9 mg/dL vs 99.9 mg/dL, P = .01)  Decrease in the proportion of subjects with LDLc levels ≥ 100 mg/dL (20% vs 50%, P=.08)  Proportion of subjects with BP values exceeding 130/80 mm HG also did not improve |
| Solberg (2006) | 17 primary care clinics  7,500 diabetes patients, 3,750 heart disease patients and 3,000 patients with depression | Group transformation process that developed and implemented change strategy based on all 6 CCM elements. | All measured at two time points – prior to implementation of the CCM and then again after implementation  Assessing Chronic Illness Care Survey  Diabetes  Glycated haemoglobin  Lipoprotein cholesterol  Coronary heart disease  Lipoprotein cholesterol  Cardiac event in previous year  Depression  % patients with new diagnosis  % patients still taking anti-depressants  % patients with no follow up care | Assessment of implementation of CCM Elements in setting:  Overall scores improved during the intervention period.  Those scores for delivery DSD and SMS changed the least and were not significant  Overall change represents a 24% improvement in CCM implementation although considerable variation among the clinics.  Assessing Health Outcomes:  Diabetes quality of care improved significantly  Heart Disease quality of care improved significantly  Unable to find evidence that suggests a relationship between the measures of CCM implementation and quality improvements for patients with these conditions  The elements of DS and CIS are the only ones to show any relationship to care improvement. |
| Wang (2004) | 2 primary healthcare case studies provided | Diabetes collaborative which developed evidence based systems orientated diabetes care program for primary care services. Involved the implementation of 6 CCM elements using plan-do-study-act methods. Program offers intensive training in the CCM and quality assurance. Also a focus on sustainability. | Not provided | Case study one: A1c level at baseline 9.4 current (March 2003) value 7.9 maintained for a 3 year period  Case study two: A1c level at baseline 6.9, average 6.8 as of January 2004. Number of patients with self-management goals increased |
| Weeramanthri (2003) | Primary care sites in NT – numbers not provided | Implemented by each health service separately but able to draw on detailed CD management protocols  Computerized Information system or a standardized paper based system  Advanced Skills Course in CD Management  Community based and regional workshops on CD management  CD Operational Management Group  Preventative CD System Coordinators | Birth weights  Immunisation  Renal dialysis treatments  Diabetic amputations  Food supply  Alcohol consumption  Adult smoking  Number of eye reviews  Number of patients with angiotensin converting enzyme inhibitors  Glycated haemoglobin levels | Improvement of birth weights  Sustained high levels of immunisation  Slowing in the rate of growth of renal dialysis treatments  Decline in the number of diabetic amputations in the Top End  Improvements to the food supply  Relatively stable per capita alcohol consumption  Continuing decline in adult smoking prevalence  40% of patients had an eye review in the previous 2 years (up from 20% five years previously)  70-80% of patients were on angiotensin converting enzyme inhibitors (up from 30% five years previously)  40 – 80% in different communities had a glycated haemoglobin level measured in the previous six months (compared to less than 15% in previous 6 months) |
| Wellingham (2003) | COPD RCT study – 130 (78 intervention, 52 control) patients  CHF study – Not provided  Diabetes – Not provided  Asthma – Not provided but noted a low response rate | Plan Do Study Act cycle used to develop culturally competent systems and provider skills., information systems, selection of target groups of patients, clinical guidelines and education of patients and providers  Support from and linkage to secondary care – services and advice  Skills in behavioral change, patient care planning  Practice systems that encourage proactive care  Evaluation, audit, feedback | COPD – Respiratory Bed Days  CHF – number of echocardiograms received by patients, prescribing adherence to guidelines, secondary care utilisation  Diabetes – HbA1c levels, provider behaviour | COPD – There was a significantly greater reduction in respiratory bed days (mean 2.6 days, 95%CD (0.5, 4.7)) for the intervention group compared with the control group. A pulmonary rehabilitation program was attended by 60^of those in the intervention group and 10% of those in the control group  CHF – The number of echocardiograms received by patients increased and prescribing became more consistent with guidelines. A significant reduction in secondary care utilization occurred.  Diabetes – reduction in HbA1c levels, and clear change in provider behavior, though no formal analysis was published |
